# Croakers

**Published:** 1874-09

**Authors:** 


### CROAKERS.

Of all the poor, miserable, unfortunate, unhappy beings in this world, the constitutional croaker is the highest type. He finds fault with everything; and insists upon it, that the world and everybody in it is going to ruin with telegraphic speed. According to him, "downwards" is the order of the day. He confidently asserts that men are degenerating, are growing smaller; that the human constitution is becoming impaired; that effeminacy is spreading everywhere; that the stamina of our wives and daughters is rapidly deteriorating with each successive generation, and that before long child bearing will cease for want of physical vigor; that in short the men are becoming pigmies, the women are wilting and withering away, and that the world will be depopulated in a very short time, unless something is done. Then he takes another tack, and vigorously declares that the morals of the people are degenerating, that social disorders are increasing with great rapidity; and as for honesty, it has clean gone out of the world; that every business man would become a swindler, only if he was sure it would never be found out—judging from himself. But what are the plain facts of the case. In the days of the first Napoleon it was necessary that every man who entered the army should measure considerably over five feet, but as men perished in battle the army standard was constantly lowered, until it was a rare thing to meet a Frenchman under forty who was not a Lilliputian, and the outside world began to take up the impression that France was full of pigmies; but since then the average height of the French has increased some two inches.

Two hundred years ago, the average life of an Englishman was less than thirty years; now, it is over forty-one. King David was only seventy-five years old; Solomon a little over fifty. Said the Psalmist: "The days of our years are threescore years and ten; and if by reason of strength they be fourscore years, yet is their strength labor and sorrow, for it is soon cut off and we fly away;" evidently seeming to think that seventy or eighty years was a long time to live. Only last week sixty persons died in Philadelphia, all of whom were over eighty years of age. The average age of the inhabitants of London has been steadily increasing for two hundred years.

The old folk to me they say
The times grow worse from day to day.
But I say no!
I put it so:—
The times are just the times we've always had,
It is the people who have grown so bad.

—*Seefeld.*

As for child-births they are increasing in number every year, and the total population of the globe is several hundred millions more than it was five hundred years ago.

That the intellect of the race is becoming more exalted, and that the grasp of the human mind is wider and more outsearching, is demonstrated in the great "Expositions" of London and Paris and Vienna, as well as by the locomotive, the steam-ship and the telegraph. Everywhere there is human development, and progress and improvement are the watchwords of the age. As to business integrity, no higher types of it can be found on the globe than in New York city. A few men have been found who were unfaithful to their trusts, but what are these to the multitude of merchant princes and men of all trades and callings, who have held fast to their integrity in these perilous times when millions of money have been lost. The merchants have slaughtered their goods; mechanics and tradesmen have sacrificed all that they could lay their hands on and disposed of their property for what they could get for it, in their anxiety to pay their debts; it is a notorious fact that when the panic was at its worst, and brokers had overdrawn their bank accounts to the extent of millions on millions, just one single man of them, in this great New York, failed to make good his bank account in the prescribed time; if a few failed to do so, it did not amount to a tenth of one per cent.

Down with the croakers, with all fault finders and prophets of evil tidings, such persons are enemies of their kind, enemies to progress, to improvements, and to all that is calculated to elevate and happify the race; they carry with them blighting and mildew wherever they go; they throw a spell over all joyousness, and clouds and darkness follow after them. The croaker never does anything but croak, and all his influences are depressive.

